# Validation of a preoperative formula to estimate postoperative pelvic sagittal alignment and mobility before performing total hip arthroplasty for patients with hip osteoarthritis

**DOI:** 10.1186/s42836-023-00171-w

**Published:** 2023-04-05

**Authors:** Hiroki Tanabe, Yasuhiro Homma, Naotake Yanagisawa, Taiji Watari, Seiya Ishii, Yuichi Shirogane, Tomonori Baba, Kazuo Kaneko, Muneaki Ishijima

**Affiliations:** 1 Department of Medicine for Orthopaedics and Motor Organ, Juntendo University Graduate School of Medicine, 2-1-1 Hongo, Bunkyo-ku, Tokyo, 113-0033 Japan; 2 Department of Orthopaedic, Faculty of Medicine, Juntendo University, 2-1-1 Hongo, Bunkyo-ku, Tokyo, 113-0033 Japan; 3 Medical Technology Innovation Center, Juntendo University, Tokyo, 113-0033 Japan

**Keywords:** Total hip arthroplasty, Pelvic alignment, Pelvic mobility, Sagittal balance, Predictive formulas, Validation

## Abstract

**Background:**

Although it is important to consider pelvic alignment and mobility in the standing and sitting positions before THA, it is not known how to preoperatively predict individual postoperative pelvic alignment and mobility. The purpose of this study was to investigate the pelvic alignment and mobility before and after THA, and to develop a predictive formula using preoperative factors to calculate postoperative sagittal alignment and mobility.

**Methods:**

One hundred seventy patients were assessed. The 170 patients were randomly divided into a prediction model analysis group (*n* = 85) and an external validation group (*n* = 85). In the prediction model analysis group, preoperative spinopelvic parameters were used to develop the predictive formulas to predict the postoperative sacral slope (SS) in standing and sitting positions and ΔSS. These were applied to the external validation group and assessed.

**Results:**

R^2^ in multiple linear regression models for postoperative SS in standing, SS in sitting and ΔSS were 0.810, 0.672, and 0.423, respectively. The values of predicted and postoperative parameters were very close with no significant difference: SS in standing (33.87 *vs.* 34.23, *P* = 0.834), SS in sitting (18.86 *vs.* 19.51, *P* = 0.228), and ΔSS (15.38 *vs.* 14.72, *P* = 0.619).

**Conclusion:**

The present study showed that the pelvic alignment and mobility after THA can be predicted using preoperative factors. Although a model with higher accuracy is needed, it is important to use a predictive formula to estimate the postoperative condition before performing THA.

## Introduction

In total hip arthroplasty (THA), the optimal positioning of the acetabular cup is mandatory to prevent dislocation. In 1978, Lewinnek *et al.* reported that the acetabular component and cup should be implanted within the “safe zone”, with a cup inclination angle of 40° ± 10° and an anteversion of 15° ± 10° [1]. However, recent studies have reported that dislocation could occur even if the cup was positioned in the Lewinnek safe zone [2–4]. Abdel *et al.* reported that 58 % of dislocated THAs had a cup implanted within the Lewinnek safe zone [5].

Recent research has found that changes in the pelvic alignment consequently changed the cup angle, resulting in posture- and time-dependent alternations in spinopelvic alignment. The posture-dependent change is that the spinopelvic alignment differs between the supine, standing, and sitting positions [6–11]. In particular, the sagittal alignment of the pelvis changed about 20° from supine to standing position [12]. The time-dependent change is that the spinopelvic alignment changes after THA [13, 14]. The pelvic inclination in the supine and standing positions is significantly more posterior at 1 year after THA compared with that before THA, and the standing pelvic inclination became significantly more posterior over a 10-year period of time [13].

There is a growing interest in the preoperative evaluation of spinopelvic mobility using the sitting position [15]. In general, the pelvis tilts posteriorly from the standing to the sitting position in which the distance between acetabular and femoral side increases, which avoids impingement [12]. However, it was reported that certain patients, even if they had not undergone previous spinal fusion surgery, had a decreased posterior tilt during postural changes from standing to sitting, potentially increasing the risk of anterior impingement [16]. Furthermore, pelvic mobility, assessed as the change in sacral slope (SS) between the standing and sitting positions, varies widely among patients, and there existed an individual variation in the ΔSS after THA [17, 18].

Although it is important to consider the pelvic alignment and mobility in the standing and sitting positions before THA, it is not known how to preoperatively predict individual postoperative pelvic alignment and mobility. Therefore, it is unclear whether pelvic alignment and mobility can be predicted using preoperative factors. The purpose of this study was to investigate the pelvic alignment and mobility before and after THA, and to develop a predictive formula by using preoperative factors to calculate the postoperative sagittal alignment and mobility.

## Materials and methods

### Participants

After obtaining institutional review board approval, we retrospectively reviewed the medical records of all 498 patients who had undergone primary THA at our university hospital from May 2013 to December 2017.

**Fig. 1:**
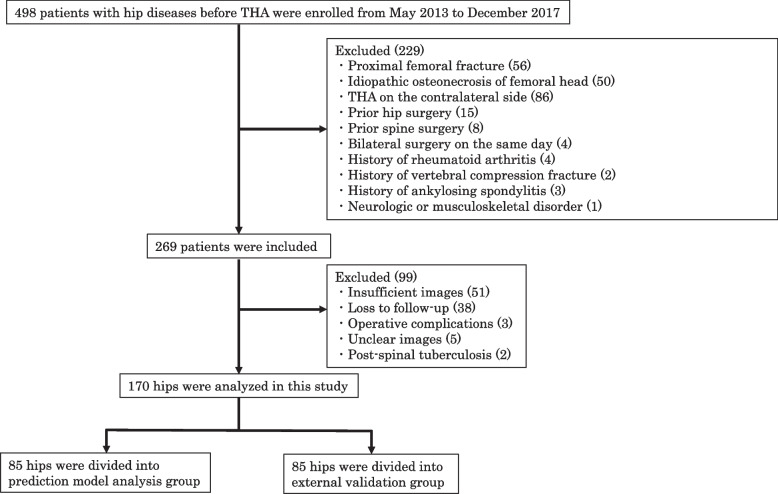
Study flow chart

The study flowchart is shown in Fig. 1. The exclusion criteria were previous hip surgeries, including THA, osteotomy, and osteosynthesis on the ipsilateral side (osteotomy, *n* = 9; osteosynthesis, *n* = 6) or THA on the contralateral side (*n* = 86); idiopathic osteonecrosis of the femoral head (*n* = 50); trauma (*n* = 56); previous spine surgery (*n* = 8); ankylosing spondylitis (*n* = 3); rheumatoid arthritis (*n* = 4); bilateral surgery on the same day (*n* = 4); neurological or musculoskeletal disorders or diseases that might adversely affect pelvic alignment (Parkinson's disease, *n* = 1); operative complications such as fracture, nerve palsy, and postoperative implant loosening (femoral fracture, *n* = 1; femoral nerve palsy, *n* = 1; cup loosening, *n* = 1); new vertebral compression fracture during follow-up (*n* = 0); limitations of ordinary activity or work after THA because of moderate or severe hip pain graded in terms of the modified Harris hip score (*n* = 1); post-spinal tuberculosis (*n* = 1); unclear images (*n* = 5); insufficient images (*n* = 51); and loss to follow-up (*n* = 38). After eliminating patients who met the exclusion criteria, the study cohort comprised 170 patients (34 men, 136 women) with hip osteoarthritis who had undergone primary THA. These 170 patients were randomly assigned into a prediction model analysis group (*n* = 85) and an external validation group (*n* = 85).

### Operative procedure

All operations were performed via the direct anterior approach, with the patient in the supine position on a standard table with fluoroscopic control [19]. A cementless stem was used in all cases, including the Accolade TMZF or Accolade II (Stryker Orthopaedics, Mahwah, NJ, USA) in 153 hips, Taperloc Complete Microplasty system (Zimmer Biomet, Warsaw, IN, USA) in five, meta-diaphyseal anchoring calcar-guided short-stemmed Optimys (Mathys Ltd., Bettlach, Switzerland) in eight, TwinSys femoral stem (Mathys Ltd.) in two, and Modulus System (Lima Corporate San Daniele Del Friuli, Udine, Italy) in two. A cementless cup was used in 166 patients; the Trident cup (Stryker Orthopaedics) was used in 157 hips (modular dual mobility cup in 54 hips), G7 cup and G7 dual mobility system (Zimmer Biomet) were used in five, and a Kerboull-type acetabular reinforcement device with X3 Rim Fit (Stryker Orthopaedics) was used in four. Postoperatively, all patients followed the same rehabilitation protocol and walking exercise with full weight-bearing began on postoperative day 1.

### Radiographic protocol

Sagittal spinopelvic alignment was assessed on standing lateral radiographs of the full spine, including the pelvis and femoral heads [20]. The radiographs were obtained on a vertical film, with a fixed distance (200 cm) between the subject and the radiographic source. Radiographic data were collected in accordance with a strict protocol. Each subject was instructed to adopt a comfortable standing position, with the fingers resting on the clavicles. This position has been described as reproducible and reliable [21, 22]. Computerized picture archiving and communication system technology (SYNAPSE; Fuji Film, Tokyo, Japan) was used.

### Measurement parameters

#### Spinopelvic parameters

**Fig. 2:**
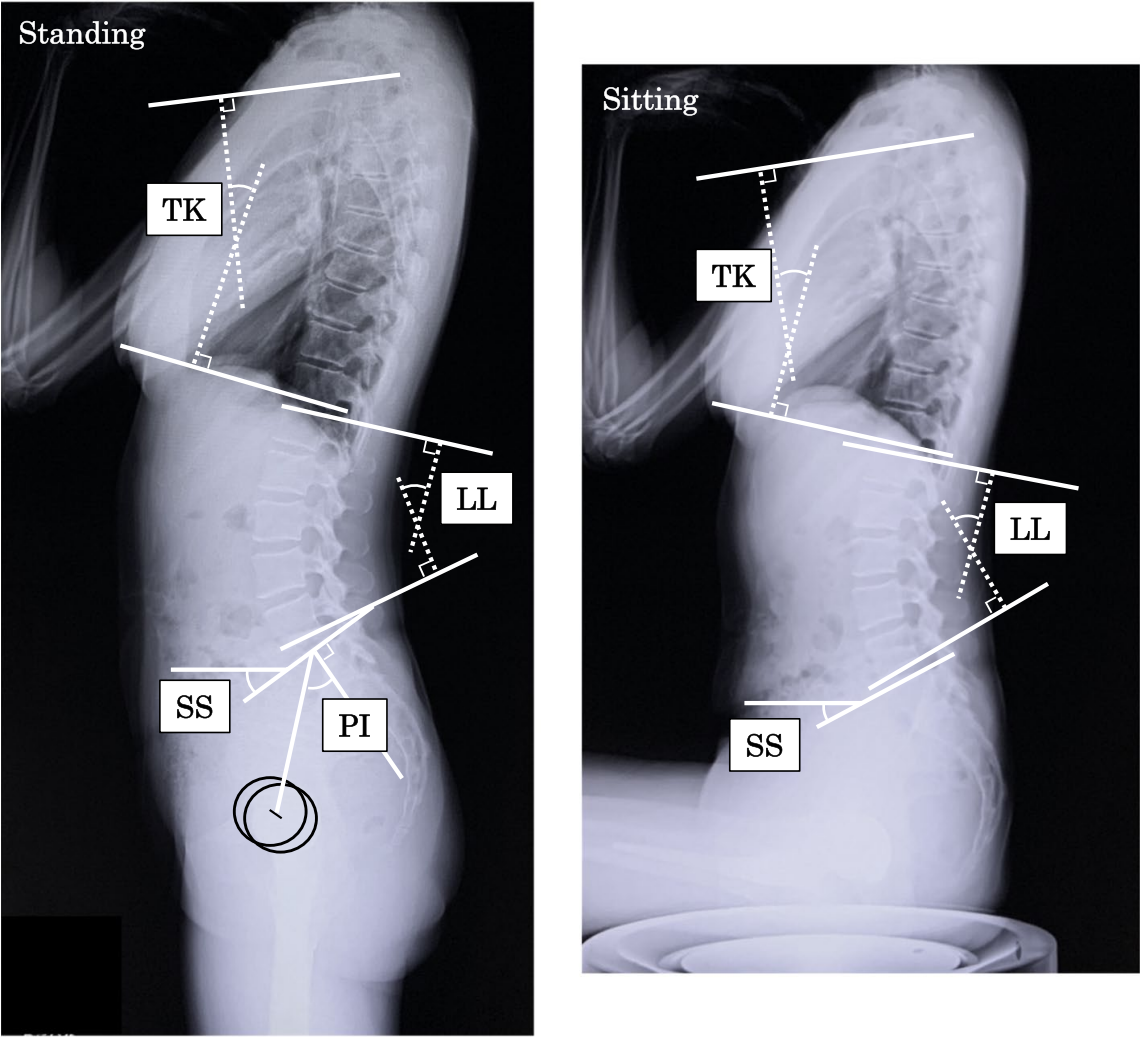
Lateral radiographs of a patient in the standing (left) and sitting (right) positions. Illustration of the spinopelvic parameters. TK, thoracic kyphosis angle; LL, lumbar lordosis angle; SS, sacral slope; PI, pelvic incidence

The following sagittal radiographic variables were measured (Fig. 2).

- ▪ Thoracic kyphosis angle (TK): the angle between lines drawn along the inferior endplate of T12 and the superior endplate of T4. Lordosis was expressed as a negative value, while kyphosis was presented as a positive value.
- ▪ Lumbar lordosis angle (LL): the angle between lines drawn along the superior endplates of L1 and S1. Lordosis was expressed as a positive value, while kyphosis was given as a negative value.
- ▪ Pelvic tilt (PT): the angle between the vertical plane and the line joining the middle of the upper endplate of S1 with the bicoxofemoral axis. PT is a positional parameter.
- ▪ Pelvic incidence: the angle between the line perpendicular to the middle of the superior endplate of S1 and the line joining this point to the bicoxofemoral axis. Pelvic incidence is a morphological parameter that is not affected by posture or pelvic position [12, 23, 24].
- ▪ SS: the angle between the horizontal plane and the upper endplate of S1. SS is a positional parameter that varies in accordance with pelvic position.

Each parameter was measured independently by one observer (S.I.). The intra- and inter-observer reliabilities of these measurements have been validated in previous studies [11, 23, 25].

#### Spine and contralateral hip disease

The following spine and contralateral hip diseases were evaluated.

- ▪ Spondylolisthesis: a slip of > 5 mm between two adjacent vertebrae on the lateral radiograph [26].
- ▪ Vertebral compression fracture: the presence of wedge, biconcave, or compression deformities [27].
- ▪ Lumbar scoliosis: the Cobb angle of > 10°.
- ▪ Osteoarthritis on the non-operative side: Tönnis grade 1–3 on anteroposterior pelvic radiographs [28, 29].

### Measurement periods

The parameters were measured a few days before THA (preoperative period) and more than 3 months after surgery (postoperative period), when the patients had achieved normal gait and kinematics [30].

### Statistical analysis

The Mann-Whitney U test was used to compare continuous variables between two groups, while the Chi-squared test was used to compare dichotomous variables. Before conducting the multivariate analysis, Spearman's rank correlation coefficient was calculated to evaluate the relationships between variables.

In the prediction model analysis group, multiple regression analysis was performed using the stepwise method, with each postoperative factor (postoperative SS in standing, postoperative SS in sitting, and postoperative ΔSS) as the dependent variable and each preoperative factor as the independent variable. This was used to develop predictive formulas that were applied to the external validation group. The predicted values in the external validation group were compared with the actual postoperative values of each parameter in the same group. Data were statistically analyzed using IBM SPSS Statistics for Macintosh, version 22.0 (IBM Corp., Armonk, NY, USA).

## Results

### Patient characteristics

The basic patient characteristics and pre- and postoperative spinopelvic parameters are shown in Tables 1 and 2.

**Table 1:** Patients’ characteristics

Number of patients	Total (*n*=170)	Prediction model analysis group (*n*=85)	External validation group (*n*=85)	*P* value
Sex (Male:Female)	34 : 136	18 : 67	16 : 69	0.701
Age (years)	66.5 ± 10.4 (39 – 87)	65.9±10.1	67.1 ± 10.7	0.452
BMI (kg/m^2^)	24.1 ± 3.9 (16.6 – 35.4)	24.4±4.1	23.9 ± 3.6	0.370
Spondylolisthesis (%)	40 (23.5%)	19 (22.3%)	21 (24.7%)	0.718
Compression fracture (%)	11 (6.4%)	5 (5.9%)	6 (7.1%)	0.755
Lumbar Scoliosis	55 (32.4%)	30 (35.3%)	25 (29.4%)	0.412
Osteoarthritis on contralateral side (%)	89 (52.5%)	48 (56.5%)	41 (48.2%)	0.282
Follow up period of radiograph (%)	11.4 ± 7.3 (3.1 – 23.9)	12.1 ± 7.7	10.6 ± 6.7	0.212

Mean ± Standard Deviation (range), *BMI* Body mass index

**Table 2:** Pre- and postoperative spinopelvic parameters

	Prediction model analysis group	External validation group	*P* value
PI (°)	49 (43–57)	50 (42–59)	0.877
Pre-operative			
SS-standing (°)	36 (29–43)	36 (29–44)	0.942
LL-standing (°)	38 (27–49)	40 (28–50)	0.755
TK-standing (°)	25 (17–34)	28 (20–35)	0.262
SS-sitting (°)	14 (4–14)	16 (8–24)	0.069
LL-sitting (°)	17 (2–28)	16 (5–27)	0.921
TK-sitting (°)	27 (18–35)	26 (20–36)	0.801
Post-operative			
SS-standing (°)	34 (26–42)	36 (28–41)	0.594
LL-standing (°)	38 (28–49)	41 (27–51.5)	0.679
TK-standing (°)	26 (17–34)	30 (21–37)	0.124
SS-sitting (°)	19 (8–26)	21 (13–28)	0.061
LL-sitting (°)	18 (5–32)	22 (9–31)	0.402
TK-sitting (°)	26 (17–32)	27 (21–36)	0.261

Median (IQR)

*PI* pelvic incidence, *SS* sacral slope, *LL* lumbar lordosis, *TK* thoracic kyphosis

### Multiple linear regression analysis

The Spearman rank correlation analysis revealed strong relationships between preoperative SS in standing and preoperative LL in standing, preoperative SS in sitting and preoperative LL in sitting, and preoperative TK in standing and preoperative TK in sitting (r > 8). Thus, preoperative LL in standing, preoperative LL in sitting, and preoperative TK in sitting were excluded from the multivariate analysis.

The dependent variables were the postoperative SS in standing, SS in sitting, and ΔSS. The independent variables were BMI, age, sex, pelvic incidence, preoperative SS in standing, preoperative SS in sitting, preoperative TK in standing, and the presence or absence of spondylolisthesis, vertebral compression fracture, lumbar scoliosis, and osteoarthritis in the contralateral hip. These parameters were employed to develop the following equations to predict each postoperative parameter (Table 3).

**Table 3:** Multiple linear regression analysis of postoperative SS in standing

	Unstandardized coefficient		Standard coefficient	*P* value	95% CI
	B	SE	β		
Post SS-standing					
Constant	14.836	4.678		0.002	5.528 – 24.143
Pre SS-standing	0.764	0.058	0.764	0.000	0.648 – 0.879
Pre SS-sitting	0.123	0.043	0.158	0.006	0.037 – 0.209
Age	-0.154	0.056	-0.141	0.007	-0.265 – -0.043
Postoperative SS-standing = 14.836 + preoperative SS-standing×0.764 + preoperative SS-sitting×0.123 – age×0.154 (*R*^2^ = 0.810).					
Post SS-sitting					
Constant	-0.703	3.206		0.827	-7.083 – 5.676
Pre SS-sitting	0.612	0.074	0.618	0.000	0.465 – 0.760
Pre SS-standing	0.335	0.093	0.264	0.001	0.150 -– 0.519
The present of contralateral hip OA	-4.729	1.868	-0.166	0.013	-8.446 – -1.012
Postoperative SS-sitting = -0.703 + preoperative SS-standing×0.335 + preoperative SS-sitting×0.612 – the presence of contralateral hip OA×4.729 (*R*^2^ = 0.672).					
Post ΔSS					
Constant	6.253	3.873		0.110	-1.455– 13.961
Pre SS-standing	0.436	0.108	0.402	0.000	0.221– 0.651
Pre SS-sitting	-0.491	0.085	-0.580	0.000	-0.660 – -0.323
The present of contralateral hip OA	5.639	2.167	0.232	0.011	1.327 – 9.951
The present of scoliosis	-4.548	2.211	-0.180	0.043	-8.949 – -0.148
Postoperative ΔSS = 6.253 + preoperative SS-standing×0.436 − preoperative SS-sitting×0.491 + the presence of contralateral hip OA×5.639 – the present of scoliosis×4.548 (*R*^2^ = 0.423)					

*SS* sacral slope, *OA* osteoarthritis

Postoperative SS in standing = 14.836 + preoperative SS in standing × 0.764 + preoperative SS in sitting × 0.123 – age × 0.154 (R^2^ = 0.810).

Postoperative SS in sitting = -0.703 + preoperative SS in standing × 0.335 + preoperative SS in sitting × 0.612 – the presence of contralateral hip osteoarthritis × 4.729 (R^2^ = 0.672).

Postoperative ΔSS = 6.253 + preoperative SS in standing × 0.439 − preoperative SS in sitting × 0.491 + the presence of contralateral hip osteoarthritis × 5.639 – the presence of scoliosis × 4.548 (R^2^ = 0.423).

### External validation

The values of predicted and postoperative parameters were very close with no significant difference. The SS in standing (33.87 *vs.* 34.23, *P* = 0.834), the SS in sitting (18.86 *vs.* 19.51, *P* = 0.228), and ΔSS (15.38 *vs.* 14.72, *P* = 0.619) (Table 4).

**Table 4:** Values of predicted and postoperative parameters and comparative analysis

	Predicted value	Post operative value	*P* value
SS-standing (°)	33.87 (±10.34)	34.23 (±12.09)	0.834
SS-sitting (°)	18.86(±9.93)	19.51 (±11.66)	0.228
ΔSS (°)	15.38 (±7.37)	14.72 (±9.67)	0.619

Mean (±Standard Deviation)

*SS* sacral slope

The match rates within the targeted error ranges between the predicted and measured postoperative values are shown in Table 5.

**Table 5:** The match rate within a targeted error range between the predicted and the measured values

Difference	Postoperative SS-standing (%)	Postoperative SS-sitting (%)	Postoperative ΔSS (%)
	Predicted minus postoperative value	Predicted minus postoperative value	Predicted minus postoperative value
< 2°	31.8	16.5	20.0
< 4°	58.8	42.3	41.2
< 6°	76.5	51.8	47.1
< 8°	85.9	63.5	58.8
< 10°	92.9	70.6	76.5

*SS* sacral slope

## Discussion

Dislocation after THA sometimes occurs even if the acetabular cup is implanted in the optimal position [2–5, 31, 32]. This is thought to result from the cup alignment alterations with postural changes from sitting to standing and over time after THA. Moreover, the ΔSS after THA (as a measure of pelvic mobility) varies widely between individuals [13]. Therefore, there is a need to predict postoperative spinopelvic alignment and mobility after THA. In the present study, we created formulas to predict the postoperative spinopelvic parameters and revealed that the predicted values calculated using preoperative parameters were close to the postoperative values.

We believe that it is important to evaluate not only preoperative pelvic mobility using spinopelvic parameters in standing and sitting positions but also the spinal alignment and condition of the contralateral hip before THA, and to predict the postoperative pelvic mobility using these parameters. The present results showed that pelvic mobility significantly decreased after THA. In the prediction formula, the ΔSS of patients with hip osteoarthritis on the contralateral side increased after THA. This might be caused by decreased hip motion and increased pelvic motion due to pain and/or stiffness of the contralateral hip. Similar findings have been reported previously, and it is believed that patients restricted their hip motion because of pain and stiffness and compensated for this by increasing their pelvic motion before THA. After THA, these hip limitations are improved and the compensatory pelvic motion decreases [6, 17]. In addition, the ΔSS of patients with scoliosis reportedly decreased after THA because patients with scoliosis had minimal spinal motion, sacroiliac joint degeneration, and remaining low pelvic mobility after THA [17]. These previous findings support the present results. In a similar study, Watanabe *et al.* reported that lower pelvic mobility and lumbar alignment were preoperative factors for low postoperative mobility [18].

Upon predicting the postoperative alignment and mobility before THA, the cup positioning and implant selection should be considered. In particular, it is important to identify patients with flexed or extended pelvic alignment in standing positions with low pelvic mobility. Patients with flexed pelvic alignment with low pelvic mobility (*e.g.*, after spinal fusion) may be at increased risk of anterior hip impingement in the sitting position and posterior dislocation. Therefore, the placement of the cup in low anteversion should be avoided [33, 34]. In contrast, patients with extended pelvic alignment with low pelvic mobility might be at increased risk of posterior hip impingement in the standing position and anterior dislocation. Hence, placement of the cup in high anteversion should be avoided. In both cases, a dual mobility cup or a large femoral head might be suitable [33, 34].

Although the present study created predictive formulas with a certain degree of validity, more accurate predictive formulas should be established in the future because the R^2^ values in the formulas to predict the postoperative SS in sitting and ΔSS were not sufficiently high compared with the formula to predict the postoperative SS in standing. This suggests that the prediction of the SS in a sitting position is affected by further unidentified parameters. There are two reasons for the relatively lower R^2^ values for the SS in sitting and ΔSS. First, although the patients placed their fingers on their clavicles in both the sitting and standing positions, the sitting posture might have varied widely among individuals. We assume that the sitting posture might be influenced by pain in the contralateral hip and spinal disease. Second, the present study used standard radiography rather than the EOS system. The visualization of the spinopelvic bones in the sitting position was relatively difficult compared with the standing position. Further understanding of the sitting position and better image evaluation are needed in future research [11, 35].

The present study has several limitations. First, the follow-up period ranged from 3 to 24 months. It may be too early to evaluate hip function at 3 months after THA, as the functional component of the Harris hip score tends to plateau at 12 weeks after THA via the direct anterior approach [36]. However, Ishida *et al.* reported that the PT changed most markedly within the first 3 months and then continued changing slowly until 1 year after THA [37]. Therefore, although further long-term studies are also needed, we believe that our follow-up period was appropriate. Second, we did not assess hip pain or range of motion. As the disease progresses and hip osteoarthritis becomes more severe, worsening of the contracture reportedly further impairs the ability of the pelvis to rotate posteriorly [31]. Third, we did not evaluate the patients’ spinal symptoms, which might influence the pelvic parameters. Although spinal symptoms are considered to exert only a small influence on pelvic parameters, further research is needed to verify this. Lastly, the exclusion criteria in this study were too strict, thereby the result is limited to specific patients. However, variations in patients’ conditions may or may not influence spinopelvic alignment and mobility. We aimed to analyze general patients without those variations. Further extended analysis with various patients must be performed in the future.

## Conclusion

The present study showed that pelvic alignment and mobility after THA can be predicted using preoperative factors for patients with hip osteoarthritis. However, a model with higher accuracy is needed. It is important to use a predictive formula to estimate the postoperative condition before performing THA.

## Data Availability

Data are available within the article.
